# COVID-19 and Adrenal Insufficiency: Unmasking the Link

**DOI:** 10.7759/cureus.47920

**Published:** 2023-10-29

**Authors:** Carlos A Marques, Patrícia P Almeida, André M Gonçalves, Volodymyr Nagirnyak, Joana Cabeleira

**Affiliations:** 1 Endocrinology, Diabetes, and Metabolism, Instituto Português de Oncologia de Lisboa Francisco Gentil, Lisboa, PRT; 2 Occupational Health, Centro Hospitalar do Médio Tejo (CHMT), Torres Novas, PRT; 3 Internal Medicine, Centro Hospitalar do Médio Tejo (CHMT), Torres Novas, PRT

**Keywords:** covid-19, adrenal cortex, autoimmune adrenal insufficiency, adrenal disorders, adrenal insufficiency (ai), addison's disease

## Abstract

We describe a case of a 28-year-old male who presented with general malaise, fatigue, anorexia, occasional epigastric pain, and vomiting a few days after a mild severe acute respiratory syndrome coronavirus 2 (SARS-CoV-2) infection. Clinical evolution led to weight loss (6 kg in six months) and craving for salty foods. Physical examination revealed dehydration, hypotension, and hyperpigmentation of the skin and mucosal surfaces. Laboratory tests demonstrated normocytic normochromic anemia, acute kidney injury, hyperkalemia, hyponatremia, and compensated metabolic acidosis. Adrenal workup allowed us to establish a diagnosis of adrenal insufficiency (AI) due to autoimmune adrenalitis, considering findings of a low cortisol and positive 21-hydroxylase antibodies (21OH-Abs), as well as high serum renin and adrenocorticotropic hormone (ACTH).

Atypical presentations and comorbidities may appear regarding coronavirus disease 2019 (COVID-19), such as the association between COVID-19 and the hypothalamic-pituitary-adrenal (HPA) axis, which may be affected in any patient with SARS-CoV-2 infection, thus making adrenal insufficiency a diagnosis to consider.

## Introduction

Adrenal insufficiency (AI) is a disorder characterized by a deficiency of glucocorticoids that is generally associated with a deficiency of adrenal androgens and, occasionally, of mineralocorticoids, caused by intrinsic diseases of the adrenal cortex (primary AI or Addison's disease), disorders that affect the adrenocorticotropic hormone (ACTH) secretion (secondary AI), or hypothalamic disorders affecting corticotropin-releasing hormone secretion (tertiary AI). The primary etiology is autoimmune adrenalitis, followed by infectious causes, mainly tuberculosis [1,2]. The insidious onset of non-specific symptoms such as fatigue and electrolyte imbalance frequently results in a significant delay in diagnosis, so specific features such as hyperpigmentation must raise suspicion [3].

Severe acute respiratory syndrome coronavirus 2 (SARS-CoV-2) is responsible for causing the coronavirus disease 2019 (COVID-19) infectious disease. Recently, there have been cases describing previously undiagnosed primary adrenal disease presenting during concurrent COVID-19 infection [4].

Our case report shows an association between COVID-19 and Addison's disease, both as a potential direct cause and as an infectious event that triggered an autoimmune process.

## Case presentation

**Figure 1:**
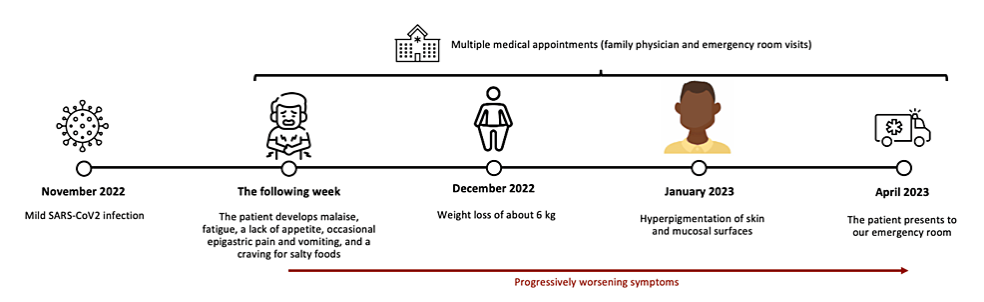
Chronologic timeline of patient's clinical evolution. SARS-CoV-2: severe acute respiratory syndrome coronavirus 2

A 28-year-old Caucasian male with no relevant personal or family history reported odynophagia and dry cough in November 2022, thus testing positive for SARS-CoV-2 infection. The following week, he developed malaise and fatigue that forced him to stop all physical activity, about 4-5 hours daily of triathlon training. Additionally, the patient presented with a lack of appetite, occasional epigastric pain and vomiting, and a craving for salty foods, which led to a consequent weight loss of about 6 kg (going from 76 to 70 kg) at around one month post-COVID-19 diagnosis. Concurrently, his family and friends started noticing the skin's darkening. During this time, the patient resorted to multiple medical appointments with his family physician mixed with emergency room visits, most times doing general blood analysis and given fluids and antiemetic treatment, with no improvement in the medium-long term. His clinical condition progressively worsened, prompting him to go to the emergency room again in April 2023, about six months post-COVID-19 diagnosis (Figure 1).

**Figure 2:**
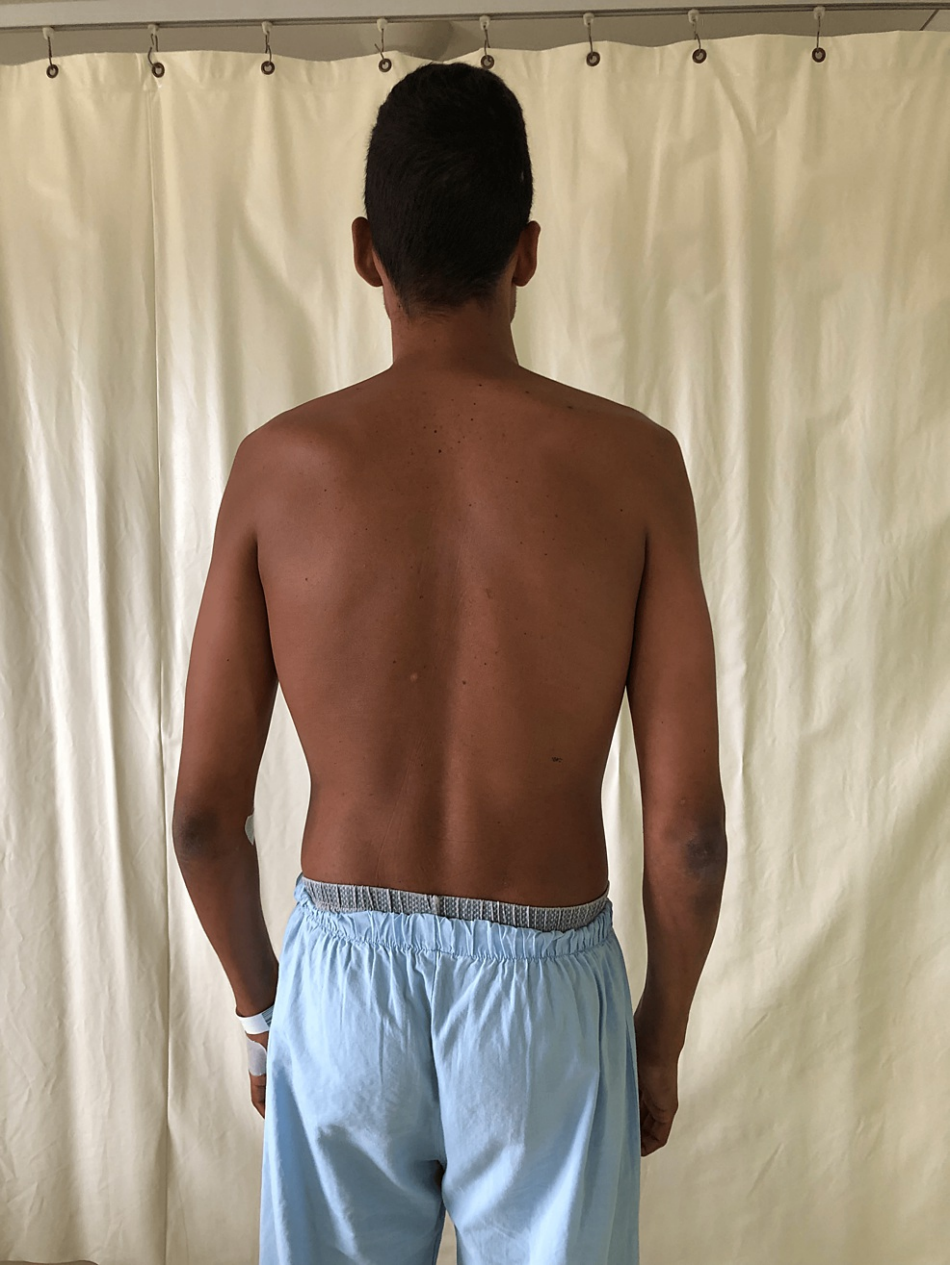
Addisonian skin hyperpigmentation.

**Figure 3:**
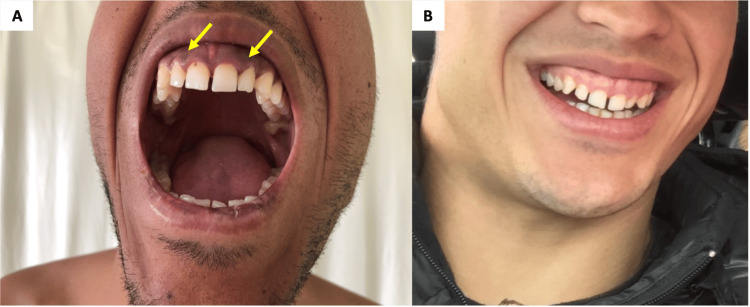
Addisonian pigmentation of the buccal mucosa (A, yellow arrows) compared to the non-altered buccal mucosa eight months prior (B).

On physical examination, the patient was dehydrated and hypotensive (blood pressure: 90/50 mmHg). A generalized hyperpigmentation of the skin and mucosal surfaces was noted (Figures 2, 3).

At this time, laboratory tests showed normocytic normochromic anemia, acute kidney injury, hyperkalemia, hyponatremia, and compensated metabolic acidosis (Table 1). A thorax, abdomen, and pelvis computed tomography (CT) revealed small adrenal glands.

**Table 1:** Laboratory results during the course of the patient's illness. NA, not applicable; HCO_3_, bicarbonate

Laboratory results		Inpatient			Outpatient	
Laboratory parameters	Reference range	Day 1	Day 5	Day 9	Day 28	Day 74
Hemoglobin (g/dL)	12.5-15.5	12.2	10.0	9.9	12.1	13.1
Mean corpuscular volume (fL)	80.0-100.0	81.6	79.7	82.6	89.8	84.6
Mean corpuscular hemoglobin (pg)	27.0-32.0	28.1	27.8	27.8	29.3	29.2
Mean corpuscular hemoglobin concentration (g/dL)	32.0-35.0	34.5	34.8	33.7	32.6	34.5
White blood cells (10^9^/L)	4.0-10.0	6.54	7.7	5.39	3.5	4.91
Platelets (10^9^/L)	150-400	244	188	192	188	178
Glucose (mg/dL)	74-106	74	192	102	104	93
Sodium (mmol/L)	136-146	127	130	138	138	134
Potassium (mmol/L)	3.5-5.1	6.2	5.4	4.0	3.9	4.4
Chloride (mmol/L)	101-109	97	104	104	105	101
HCO_3_ (mmol/L)	21.0-28.0	20.8	NA	NA	NA	NA
Urea (mg/dL)	17-43	115	93	40	48	52
Creatinine (mg/dL)	0.8-1.2	1.6	1.0	0.8	1.0	1.1

The patient was admitted to the internal medicine department for further studies.

Given that one and a half liters of 0.9% NaCl given daily failed to help both clinically and analytically, an adrenal crisis diagnosis was considered, and treatment with hydrocortisone was initiated, firstly 100 mg bolus, followed by 50 mg bolus every six hours. The patient showed progressive clinical and analytical improvement.

Studies for syndromic diagnosis, both concerning location and etiology, were performed (Table 2). Serum cortisol collected at 8 AM was low (25.07 nmol/L), raising a strong suspicion for adrenal insufficiency. In order to define the affected level of the hypothalamic-pituitary-adrenal (HPA) axis, serum ACTH was increased (2000.0 pg/mL), thus suggesting primary adrenal insufficiency. Aiming at etiological diagnosis, both anti-adrenal antibodies and autoantibodies against the steroidogenic enzyme 21-hydroxylase antibodies (21OH-Abs) were performed and came back negative and positive, respectively. Autoimmune adrenalitis was acknowledged as the final diagnosis, which is the most prevalent etiology, and presents with positive 21OH-Abs in approximately 75% of cases [2].

**Table 2:** Adrenal laboratory testing during the course of the patient's illness. NA, not applicable; ACTH, adrenocorticotropic hormone

Adrenal laboratory results (inpatient)	Normal range	Day 4	Day 7	Day 9
AM cortisol (nmol/L)	185.00-624.00	25.07	NA	NA
AM ACTH (pg/mL)	6.0-48.0	2000.0	25.2	NA
Aldosterone (pg/mL)	70.0-300.0	5.0	NA	NA
Renin (mUI/mL)	4.2-45.6	1000.0	76.4	NA
Anti-adrenal antibodies	NA	NA	NA	Negative
21-Hydroxylase antibodies	NA	NA	NA	Positive

Considering the most common infectious etiology, an interferon gamma release assay (IGRA) was performed and came back negative, excluding *Mycobacterium tuberculosis* infection.

The patient gradually improved under corticosteroid therapy with hydrocortisone dosage titrated down to 35 mg/day, apportioned into four daily doses (10 mg + 5 mg + 10 mg + 10 mg). Fludrocortisone was added at a 0.1 mg/day dosage and remained in outpatient treatment.

Patient follow-up includes a periodic appointment with therapy dosage management. At the latest follow-up (four months after AI diagnosis), he was found asymptomatic under oral maintenance therapy of hydrocortisone 20 mg/day (10 mg + 5 mg + 5 mg) plus fludrocortisone 0.1 mg/day and was able to restart physical activity with a highly functional status. No clinical complications have been reported up to date, and clinical evaluation systematically shows improving anemia and normalized renal function and ionogram (Table 1).

## Discussion

SARS-CoV-2 may affect the entire respiratory tract, and ordinary clinical signs include dry cough, fever, fatigue, and dyspnea in advanced cases [5]. Nevertheless, other organs and systems may be affected by the virus, such as the adrenal gland, as shown in other studies by the existence of adrenal viral tropism and replication in COVID-19 patients [6].

The positive-sense single-stranded RNA enters the pneumocyte using the host angiotensin-converting enzyme 2 (ACE2) as a receptor. Moreover, the enzyme is expressed in the adrenal glands' arterial and venous endothelial cells and many other organs [7], including the kidney and cardiovascular and central nervous systems [6].

In COVID-19 patients, there is no available data on cortisol release dynamics yet (scarce literature in this regard). However, existing data from outbreaks originated by different members of this virus family allows us to suggest that cortisol dynamics in COVID-19 patients are likely to be changed through various mechanisms.

The detection of the virus within the adrenal gland and the subsequent gathering of inflammatory cells, which then perish, suggest that SARS-CoV-2 directly initiates adrenalitis. Consequently, this significant damage to the gland can potentially lead to primary adrenal insufficiency, causing disruption in the HPA axis and reduced cortisol production [6].

Also in this way, coagulation disorders in COVID-19 patients may cause acute adrenal insufficiency, with some studies showing CT findings of acute adrenal infarction, widespread microthrombosis, and adrenal hemorrhage [6].

Another mechanism is that the virus expresses certain amino acid sequences through mimicking the host's ACTH. Therefore, antibodies against the virus could target it and tease a relative cortisol insufﬁciency [6].

Furthermore, severe acute respiratory syndrome coronavirus (SARS-CoV) and SARS-CoV-2 might affect the HPA axis. Leow et al. [8] first reported biochemical evidence of this connection by evaluating 61 survivors of the severe acute respiratory syndrome (SARS) outbreak three months after recovery and periodically after that. Central hypocortisolism was evident in 40% of patients, the majority of which resolved within a year. In autopsy studies, hypothalamus analysis identified edema and neuronal degeneration along with the SARS-CoV genome. As hypothalamic and pituitary tissues express ACE2, they can therefore be viral targets [7].

This HPA axis involvement can manifest through various forms besides AI, such as depression [9] or acute insomnia [10].

It is also important to notice that the involvement of the HPA axis may be transient and can resolve over time. A study found that 13.63% of COVID-19 subjects had AI at least three months after recovering from the infection. The study followed up on a subset of patients with AI after 12 months and found that the HPA axis had recovered in six out of nine subjects [11]. Urhan et al. [12] and Gonen et al. [13] have suggested the same. Overall, around 8%-16% of subjects had HPA axis involvement within three to seven months after recovery.

The striking aspect of this case is the timeline. The patient's initial SARS-CoV-2 infection occurred just a few days before the onset of AI symptoms, which raises questions about the potential link between the two events. While existing literature acknowledges the virus' presence in the adrenal gland and the possibility of direct viral damage, it is essential to recognize that the exact mechanisms behind this relationship remain a subject of ongoing research.

Through this case, we sought to establish an association between SARS-CoV-2 infection and AI as a potential direct etiology of our patient's Addison's disease or, more likely, as an infectious event that triggered an autoimmune adrenalitis process. Importantly, we also ruled out tuberculosis, a well-known infectious cause of AI, through a negative IGRA. Given that the non-specific symptoms of fatigue and general malaise followed the COVID-19 infection, the patient himself and other doctors underestimated these symptoms, considering the post-COVID-19 condition as the most probable cause. Therefore, it masked Addison's disease in evolution, which made it a challenging and lengthy diagnosis.

By the time the patient arrived at the emergency department, he already presented a clinical condition with several signs and symptoms suggestive of adrenal insufficiency. In addition, laboratory evaluation followed by an immediate improvement after starting treatment with corticosteroids contributed to achieving the final diagnosis.

The involvement of the endocrine system in COVID-19 is so relevant that an "endocrine phenotype" of COVID-19 has progressively acquired clinical relevance. However, while the contribution of endocrine dysfunction to the severity and outcomes of COVID-19 does not remain fully clarified, the impact of SARS-CoV-2 on the endocrine system may be severely underreported due to the lack of awareness of the public and clinicians [14].

## Conclusions

As time progresses and we learn more about COVID-19, it is important to be aware of atypical presentations and associated comorbidities. Through this case, we aimed to highlight the association between COVID-19 and the HPA axis. The possibility of it being affected in any patient with SARS-CoV-2 infection makes adrenal insufficiency a diagnosis that should be considered.

Therefore, considering the adrenal glands' role in stress response and immunoregulation, this finding highlights the importance of monitoring the HPA axis in COVID-19 patients, both in acute stress situations and in recovery.
